# INDUSTRY–ACADEMIA COLLABORATION PROJECT FOR CREATION OF A LEARNING HEALTH SYSTEM FOR OLDER CARE: A CASE STUDY

**DOI:** 10.1093/geroni/igae098.2861

**Published:** 2024-12-31

**Authors:** Keiko Ishii, Maiko Noguchi-Watanabe, Sakiko Fukui

**Affiliations:** Tokyo Medical and Dental University, Bunkyo-ku, Tokyo, Japan; Tokyo Medical and Dental University, Bunkyo-ku, Tokyo, Japan; Tokyo Medical and Dental University, Bunkyo-ku, Tokyo, Japan

## Abstract

The study aimed to establish a learning health system wherein artificial intelligence, the Internet of Things, and healthcare professionals collaborate to promote the well-being of older adults, families, and the healthcare professionals involved in older care. This case study seeks to elucidate the roles and challenges encountered by researchers within this industry- academia collaborative project. By employing the Project Management Body of Knowledge’s (PMBOK’s) 12 management principles (stewardship, team, stakeholders, value, system thinking, leadership, tailoring, quality, complexity, risk, adaptability and resiliency, change) as the analytical framework. As a preliminary step toward the main project, a joint research agreement with the relevant company in January 2022 was concluded, a comprehensive cooperation agreement in October of the same year, and commenced the project in April 2023. The project team comprised three researchers (home care nursing), four staff members (open innovation center and Industry-Academia Collaboration Division) from the university, and two employees from the company. The researchers participated in monthly meetings and proposed a research plan and strategies to utilize the research outcomes of the new company. To determine the number of visiting nurse users, we calculated the expected number of users at our university hospital. We also discussed the variables to be utilized in the algorithm development. An analysis based on the 12 principles reveals that although researchers tended to implement values and systems thinking, they had difficulties with other members regarding team and leadership. This research provides insights for project implementation for researchers promoting collaborative industry-academia research.

